# A Review of the Motion Planning and Control Methods for Automated Vehicles

**DOI:** 10.3390/s23136140

**Published:** 2023-07-04

**Authors:** Xiaohua Song, Huihui Gao, Tian Ding, Yunfeng Gu, Jing Liu, Kun Tian

**Affiliations:** 1 School of Electronic Information Engineering, Xi’an Technological University, Xi’an 710021, China; sxh831018@163.com (X.S.); ghh816ghh@163.com (H.G.); 17609235664@163.com (T.D.); g735149193@163.com (Y.G.); tiankun.xtu@foxmail.com (K.T.); 2 School of Marine Science and Technology, Northwestern Polytechnical University, Xi’an 710072, China

**Keywords:** motion planning, automated vehicles, tracking control

## Abstract

The motion planning and control method of automated vehicles, as the key technology of automated vehicles, directly affects the safety, comfort, and other technical indicators of vehicles. The planning module is responsible for generating a vehicle driving path. The control module is responsible for driving the vehicle. In this study, we review the main methods and achievements in motion planning and motion control for automated vehicles. The advantages and disadvantages of various planning and control methods are comparatively analyzed. Finally, some predictions and summaries based on the existing research results and trends are proposed. Through this analysis, it is believed that various types of algorithms will be further integrated in the future to complement each other’s strengths and weaknesses. The next area of research will be to establish more accurate vehicle models to describe vehicle motion, improve the generalization-solving ability of algorithms, and enhance the planning and control of integrated ‘human-vehicle-road’ traffic systems.

## 1. Introduction

Automated vehicles, also referred to as smart cars or driverless cars, are vehicles that can operate without human intervention using sensors to gather information about their surroundings and generate decision commands to execute driving behaviour. Automated vehicles are highly integrated and complex systems that incorporate modern sensing, information, and communication technologies, automatic control technologies, and artificial intelligence. These high-tech components work together to create an intelligent body for the vehicle. An autonomous driving system is essentially a result of learning, understanding, imitation, and optimization of the three crucial components of the driving process, namely, environment perception, decision planning, and control execution. These components are based on the operational experiences of human drivers [1,2,3,4]. The perception link serves as the foundation of the technology chain, providing real-time data to the intelligent driving system via constant interactions with the surrounding environment. These data include information from onboard sensors, such as vehicle cameras, LIDAR, millimeter wave radar, and positioning systems, as well as real-time road condition data obtained via vehicle–road collaboration and 5G technology. Decision planning aims to utilize environmental sensing to integrate and analyze vehicle–road environment information. This information is then used to obtain the next driving behaviour instruction, which includes both the horizontal and vertical behaviour decision planning of the vehicle. The control execution segment controls the target vehicle based on the results of upper-level planning. To ensure quick and accurate trajectory tracking, the vehicle actuators should have excellent dynamic performance and good system robustness. Currently, automated vehicles operate at level L4, which means that the vehicle operates without human intervention, but only in limited scenarios.

Vehicle motion planning involves generating a geometric path that connects the start and end points of the vehicle, while also providing information on the vehicle’s velocity along the path. This process must also ensure that the vehicle satisfies various constraints, such as kinematic/dynamic constraints and collision avoidance constraints, as well as temporal and/or spatial constraints from the internal system or external environment. However, in practical engineering applications, motion planning is typically divided into a two-stage computational architecture of path planning followed by motion timing. First, the geometric curve of the travel is generated, and then the speed along the planned curve is considered [5].

In the realm of automated vehicles, path planning plays a vital role in the decision-making process. This involves making intelligent decisions to exit the driving task and plan a feasible route based on the information gathered by the vehicle’s sensing devices, including data on surrounding pedestrians, vehicles, and buildings [6]. The path planning module can be broken down into two parts: global planning and local planning. The global path planning module generates a macroscopic path based on the start and end points, but it does not take into account the real-time environment of certain vehicles. The local route planning module of the automated vehicle generates a route with traffic and avoidance functions based on real-time environment information for the control module to follow [7,8,9].

Motion control refers to the process by which an intelligent driving system makes decisions based on the current position, posture, and vehicle speed, among other factors, using a specific logic; the system then sends commands to the throttle, brake, and steering actuators [10,11]. The two main control objectives of motion control are longitudinal control and lateral control, which aim to effectively manage the vehicle’s longitudinal and lateral movements, respectively [12]. Longitudinal control in vehicle control refers to the management of the accelerator and brake to maintain a specific speed, whereas lateral control involves using the steering system to guide the vehicle along a predetermined path [13]. Various control methods have been applied by researchers to construct models for a strongly coupled system of vehicle motion nonlinearity and tire mechanics nonlinearity in lateral control.

This paper provides an overview of the research on the planning and control of automated vehicles. The first section discusses the various vehicle motion planning methods and evaluates their pros and cons. The second section covers the research history and current status of motion control methods for automated vehicles, both domestically and internationally. Additionally, the advantages and disadvantages of the tracking control methods are evaluated. Finally, the paper concludes with an outlook on the future development of planning controls for automated vehicles.

## 2. Automated Vehicle Motion Planning

### 2.1. Graph Search Algorithm

**Figure 1:**
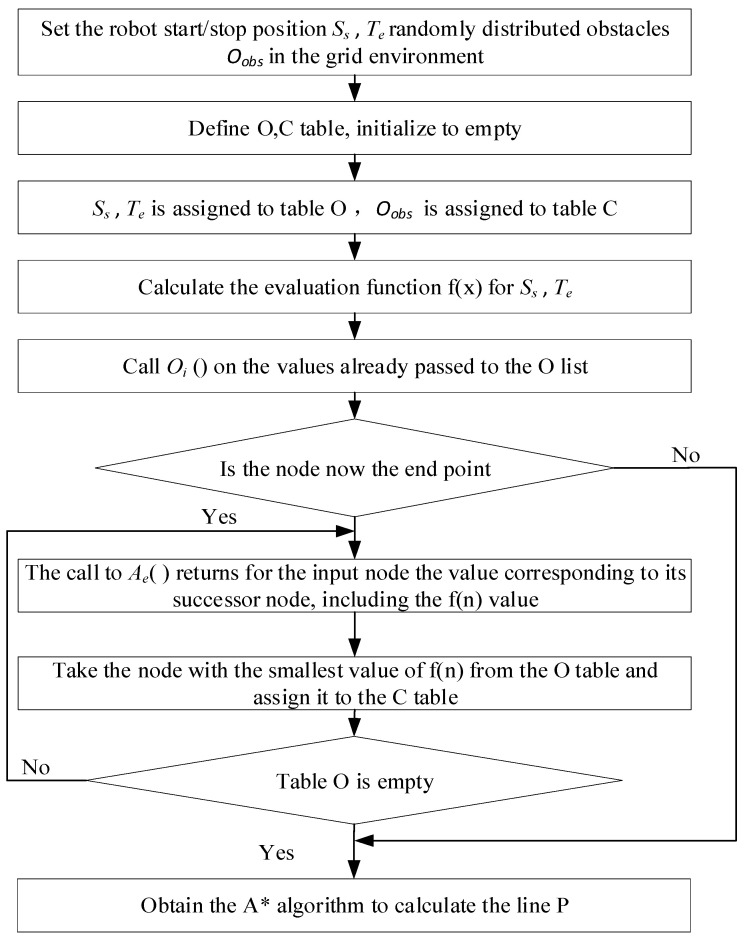
A flowchart of the A* algorithm.

The graph search algorithm is used to approximate the motion construction space of a vehicle by dividing it into feasible and infeasible grids based on the regions’ feasibility. The algorithm then searches for paths that meet the driving conditions in two stages: constructing a graph and searching for pathways within it. The visual graph method [14], the Voronoi graph method [15], and the raster decomposition method [16] are the main methods used for graph construction. In graph theory, Dijkstra [17,18] and A* [19,20,21] algorithms are popularly used to explore paths in graphs. Dijkstra is a path search algorithm that is used for finding the shortest path from a single source node. It is often used as a foundational method for solving the shortest path searching problems. On the other hand, the A* algorithm is an exploration method that is based on graph heuristics. It is commonly used to solve search tasks in rasterized maps, as the algorithm’s distance function is incorporated in the definition phase. Figure 1 shows a flowchart of the A* algorithm. However, the accuracy of the planned trajectory depends on the resolution of the map raster. A finer map raster will provide a more accurate trajectory planning, but it will also increase the computational load. Therefore, the graph search algorithm may not be suitable for complex environments. The algorithm generates trajectories that travel close to obstacles, which leads to a high-risk factor during vehicle driving. Moreover, it is less used in autonomous driving systems [22].

### 2.2. Curve Interpolation Algorithm

To tackle the challenge of computationally intensive graph search methods, scholars have proposed a curve interpolation approach [23,24]. The core idea of this method is to generate paths with high continuity and smoothness, which are subsequently fitted with road point data that satisfy vehicle travel constraints [25]. The selection of the nodes plays a crucial role in this process. Multiple sets of curves are fitted based on these nodes, and the optimal curve is selected based on specific requirements or roadblocks in the problem being addressed.

**Figure 2:**
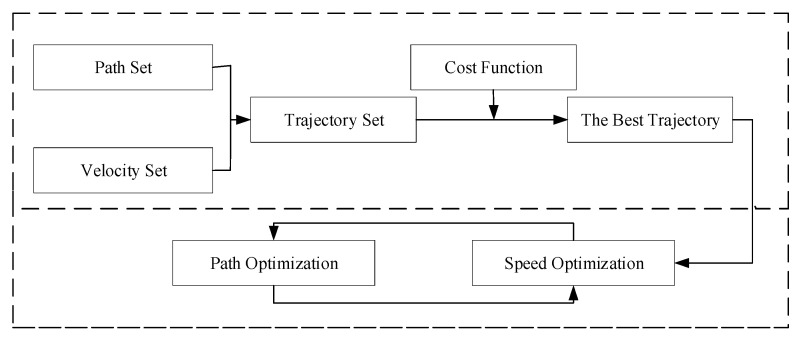
Two-part trajectory planning framework.

Polynomial curves have the more commonly used geometric relations in this type of method, which often relies on the selection of the appropriate geometric order to plan paths that meet specific kinematic constraints. For example, Xu et al. [26] presented a motion planner that utilizes polynomials to iteratively optimize the velocity and path for real-time optimization. This process is shown in Figure 2. Glaser et al. [27] used quadratic and quintuple polynomials for lateral and longitudinal planning of vehicles, respectively. Petrov et al. [28] used cubic curves to plan the path of a vehicle overtaking event. Yang et al. [29] used cubic polynomials to plan the path of an automated vehicle changing lanes, and the constraints of the surrounding vehicles were considered when the vehicle changed lanes.

The curve interpolation method is easily traceable. It has a low computational cost and high real-time performance. It is commonly used for smoothing trajectories in combination with other planning methods.

### 2.3. Sampling-Based Approach

**Figure 3:**
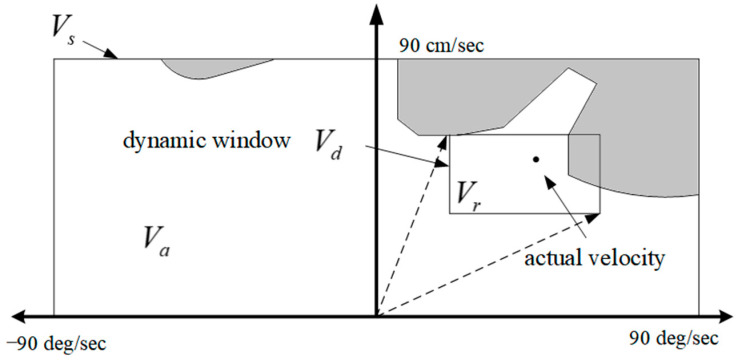
Dynamic window.

The sampling-based planning methods first need to decide on the solution space, and after dense sampling of the path points in the solution space, a path search from the starting point to the target point is performed through the effective connection between the sampled points. One of the more commonly used methods is the probabilistic roadmap method (PRM) [30], the rapidly exploring random tree (RRT) [31], and the dynamic window approach (DWA) [32,33]. The DWA algorithm is mainly used in robot path planning and low-speed self-driving scenarios [34]. The core of the DWA algorithm lies in sampling and dynamic planning. Sampling mainly includes velocity sampling in the temporal dimension and path sampling in the spatial dimension [35,36]. A schematic diagram of the dynamic window method from the literature [36] is presented in Figure 3.

The RRT algorithm has been utilized in the path planning of automated vehicles to address issues such as path curvature discontinuity and path non-optimality. To improve upon this, Karaman and Frazzoli introduced the RRT* algorithm [37], which guarantees asymptotic optimality and has been further optimized for both calculating feasible solutions and enhancing complexity and stability.

The sampling-based path planning algorithm is a fast and versatile method that considers multiple constraints. However, the accuracy and quantity of sampling greatly affect the quality of the generated paths. In complex traffic scenarios, this may result in path generation failure or require a significant amount of computational resources.

### 2.4. Artificial Potential Field Method

**Figure 4:**
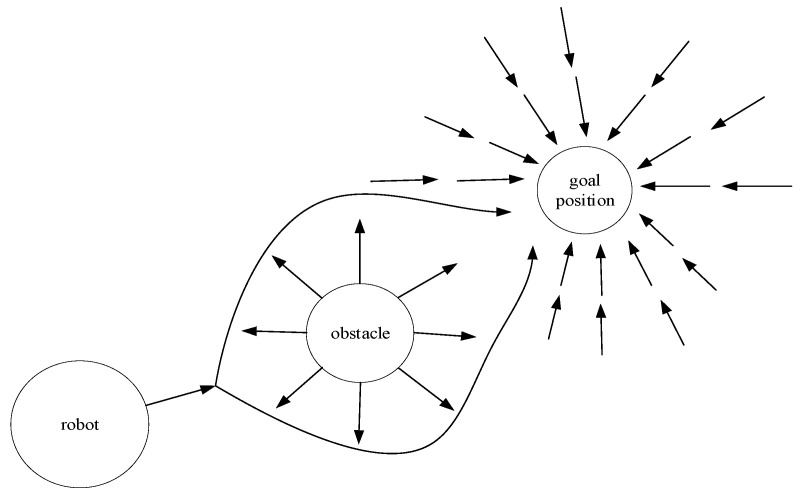
The concept of the artificial potential field method.

Khatib proposed the artificial potential field method in 1986, which models the movement of a vehicle in an environment as being influenced by a virtual force field. This method generates repulsive forces between all obstacles in the environment and the vehicle, whereas the goal point generates an attractive force. By searching for directions where the potential field weakens, feasible paths can be planned [38,39,40,41,42]. The overall planning concept is shown in Figure 4. Wahid [43] utilized an artificial potential field method to design a collision avoidance motion planning system. The system was implemented via a combination of a steering control system and a longitudinal distribution system, which enabled the vehicle to track the required motion. In their study, Wang et al. [44] examined the obstacle avoidance behavior of human drivers and used this information to construct an obstacle avoidance safety model. They also improved the artificial potential field method by reconstructing the repulsive field range associated with the obstacles. This resulted in the creation of a collision-free path that is suitable for automated vehicles. The artificial potential field method is a valuable tool for path planning in uncertain and dynamic environments. However, it is important to consider its limitations, such as its tendency to become stuck in the local minima, which can hinder its effectiveness. Additionally, the conventional APF algorithm may prevent the robot from reaching the target point due to obstacles or the influence of repulsive forces. This can make it difficult for the robot to bypass the obstacles and reach the target point.

### 2.5. Machine Learning Method

In path planning tasks that involve machine learning methods, the model takes in information about the current state of the vehicle and its surrounding environment as input and generates a planned path as output. Several machine learning techniques, such as convolutional neural networks [45,46], long- and short-term memory networks [47], reinforcement learning [48,49], and Hidden Markov decision processes [50] have been utilized for path planning in vehicles. These techniques possess remarkable advantages, including their robust learning capability, ability to predict samples for planning, and fast computational speed. Due to the complexity of traffic environments, it is not possible to achieve complete sample coverage, and there may be limitations.

### 2.6. Numerical Optimization Algorithm

In the context of self-driving, path planning can be viewed as a multi-objective optimization problem with constraints. As such, numerical optimization methods, such as the model predictive control (MPC) algorithm, are frequently utilized in path planning [51]. Optimal control, another name for numerical optimization, is also a prominent planning algorithm [52]. The MPC obtains the vehicle’s differential equations of motion in the form of a rolling optimization and solves the optimization task by taking into account the environmental and vehicle kinematic constraints [53,54].

Algorithms that are based on numerical optimization techniques enable the integration of multiple objectives in the design process and also take into account the constraints of the vehicle. This results in obtaining higher-quality solutions [55,56]. The vehicle motion planning problem typically involves a non-convex problem with high dimensionality, which can result in local minima and suboptimal solutions. Furthermore, real-time performance is an area that requires improvement in these algorithms. The algorithms that rely on numerical optimization can be utilized for post-processing paths. The outcomes of other planning algorithms can serve as the initial conditions for path optimization.

This section presents an overview of different path-planning methods and compares their effects in Table 1. Each algorithm has its own scope of application, and it is recommended to fuse various algorithms for future applications. However, to achieve more precise vehicle motion planning, it is necessary to combine the vehicle dynamics model, which can significantly increase the computational effort and make it challenging to consider the dynamic constraints. Motion planning in autonomous driving technology is a complex task that presents numerous challenges. These challenges include data processing, robustness, real-time performance, safety assurance, and practical feasibility. To overcome these challenges, it is necessary to consider various factors to comprehensively and continuously optimize and improve the technology. Only then can we achieve a safer, more efficient, and reliable autonomous driving technology.

## 3. Tracking Control Methods for Automated Vehicles

As one of the main components of an automated vehicle, path tracking is the control of the vehicle to follow a planned path with minimal deviation. The key to designing tracking algorithms is to achieve accurate and stable path tracking. Currently, the main control methods are PID control, robust control, sliding mode control, fuzzy control, pure pursuit control, LQR control, and model predictive control.

### 3.1. PID Control

**Figure 5:**
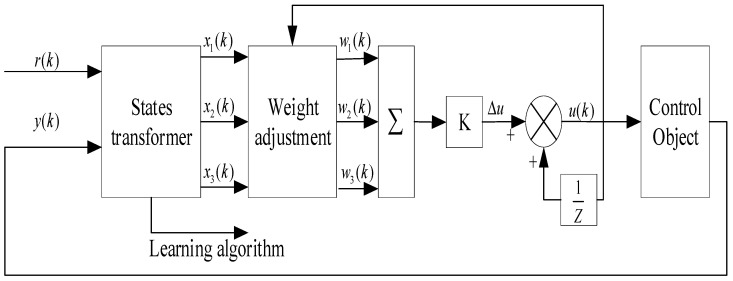
The block diagram of the single neuron-adaptive PID controller.

PID controllers are widely used in the industry due to their easy implementation and high stability [57]. However, when it comes to self-driving, conventional PID control faces challenges such as difficult parameter optimization and poor control performance. These challenges arise due to the constraints of the vehicle model, instability of the surrounding environment, and non-integrity constraints [58]. Zuo et al. [59] developed a path-tracking control method using a single neuron-adaptive PID to address issues related to frequent control oscillations. They also included a reasonable dead band to ensure accurate front-wheel corner control. The construction of their controller is shown in Figure 5. Similarly, Marino et al. [60] proposed a nested PID control method to tackle the problem of vehicle steering control.

PID control is a commonly used method in engineering, but it has inherent disadvantages in vehicle lateral control due to the coupling of lateral motion and lateral pendulum motion in a multi-state system. Furthermore, the wide range of changes in the vehicle’s longitudinal speed results in significant changes in the system’s time-varying parameters. As a result, it is relatively challenging to ensure the effectiveness of PID control in multi-variable and time-varying systems.

### 3.2. Robust Control

**Figure 6:**
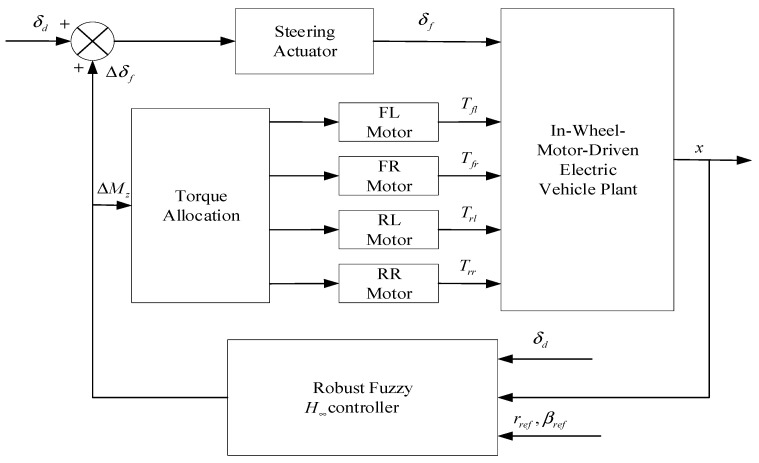
The vehicle control system architecture.

The robustness of a control system generally refers to the ability of the system to maintain some of its original qualities when its parameters or structure undergo fluctuations. Robustness includes robust stability and robust performance [61]. To enhance the immunity of path-tracking control, robust control uses methods such as frequency shaping [62] and loop shaping [63] to enable the control system to achieve the desired performance under poor operating conditions. Kayacan et al. [64] proposed a novel robust control approach that utilizes path-tracking errors and integrates feedforward and robust control techniques to guarantee precise linear and curvilinear tracking. A linear model predictive controller was developed for this purpose. Jin et al. [65] developed a robust fuzzy controller to address the challenges of nonlinearity in vehicle lateral motion. The controller utilizes the Takagi–Sugeno (T-S) fuzzy modelling approach and its construction is illustrated in Figure 6. Hu et al. [66] proposed a stable and robust yaw moment control method that effectively addressed the challenges posed by side wind disturbances and parameter fluctuations. This method was found to significantly improve the stability of the model. Robust control is often successful in mitigating disturbances, but achieving optimal control can be challenging.

### 3.3. Sliding Mode Control

Sliding mode control (SMC) is essentially a distinctive type of nonlinear variable structure control, introduced by the Soviet scholar Utkin in the 1950s, and the method’s nonlinearity is manifested in the discontinuity of the control [67]. A unique aspect of this control strategy is that the system’s structure is not static and can be intentionally modified during the dynamic process to adhere to a predetermined ‘sliding mode’ for the system.

**Figure 7:**
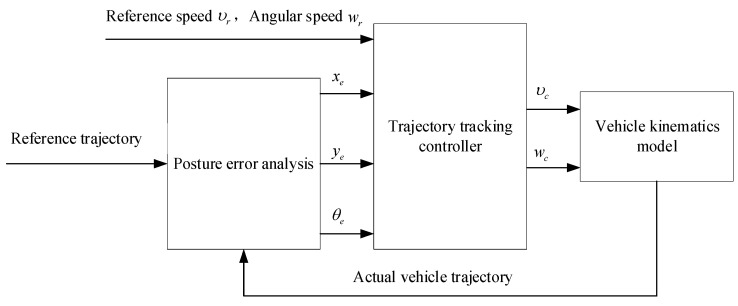
The algorithm process.

According to academic research, the sliding mode design is decoupled from object parameters and perturbations, which results in several benefits such as rapid response, robustness to parameter changes and disturbances, and simple and reliable control action. This information is supported by reference [68]. Jiang et al. [69] modelled the lateral dynamics of the vehicle and designed an SMC that was able to enhance the vehicle’s tracking performance of the desired path, and the whole process of the controller algorithm is shown in Figure 7. Wang et al. [70] presented an SMC method that utilized the convergence law to enable vehicles to accurately and efficiently follow a desired path with smooth movements.

Sliding mode control (SMC) was utilized to handle the uncertainty of the system, resulting in an accelerated convergence speed of the system state and a limited jitter phenomenon. SMC is a reliable method for controlling uncertain systems due to its ability to effectively deal with uncertainties and disturbances. Wang et al. [71] presented an SMC approach that utilized a nonlinear vehicle dynamics model with seven degrees of freedom. The proposed controller was capable of effectively guiding the vehicle to follow the desired yaw rate and path with stability. Simulations and experiments showed that the path-tracking deviation fluctuates less with changes in vehicle speed and road curvature, and the controller has better robustness and adaptability.

The sliding mode control has several advantages, such as its ability to overcome system uncertainty, strong robustness to disturbances and unmodelled dynamics, and a good control effect, particularly for nonlinear systems. However, when the state trajectory reaches the sliding mode surface, it becomes challenging to slide strictly along the surface towards the equilibrium point. Instead, the trajectory may cross back and forth on both sides, causing jitter as it approaches the equilibrium point.

### 3.4. Fuzzy Control

Fuzzy control is a contemporary approach to automatic control theory that utilizes the principles of fuzzy mathematical theory, which includes fuzzy set theory, fuzzy linguistic variables, fuzzy logic reasoning, etc. Fuzzy control is based on sensor technology, computer technology, and automatic control theory [72,73,74]. The structure of a fuzzy controller typically consists of four key components: fuzzification, knowledge base, fuzzy inference, and clarity. It does not require precise system modelling and is highly fault-tolerant [75,76]. Masmoudi et al. [77] proposed a PI control algorithm that utilized fuzzy logic to achieve the path tracking of omnidirectional robots. By adjusting the PI system parameters to reduce the dynamic changes of the target and the planning errors, the robot can avoid obstacles and reach the end point smoothly. Tang et al. [78] developed a fuzzy control algorithm-based lateral controller for automated vehicles and compared its performance with the MPC lateral controller and the optimized MPC lateral controller that utilized the PSO algorithm. According to the experimental results, the fuzzy control algorithm was found to be appropriate for operating conditions that involve low and medium speeds, and the accuracy under high-speed operating conditions is inferior to that of the MPC algorithm. Diao et al. [79] created a vehicle dynamics model and suggested a dynamic two-point pre-scan strategy. This strategy was used to design a fuzzy controller, which was then used to establish a lateral error model. Using simulation tests, the authors demonstrated that this control strategy was effective for path tracking on roads with large curvatures. Guo et al. [80] proposed a switching controller that utilizes both optimal control and fuzzy control. The optimal control was applied when the vehicle was turning at small angles, whereas the fuzzy control was used when turning at large angles. This approach addressed the issue of mismatching of the controller model caused by the strong nonlinearities that arise in the vehicle during large lateral acceleration conditions. Fuzzy control offers an advantage over traditional control methods as it does not require an exact mathematical model of the controlled object. This is particularly useful in situations where the controlled object is complex or difficult to model mathematically. The use of fuzzy control can be highly effective in situations where a system is subject to uncertainties or disturbances. This makes it a valuable tool in various fields of engineering and science. Specifically, in the case of automated vehicles, which are complex systems with multiple degrees of freedom and coupled mechanical properties of their components, fuzzy controllers can provide effective lateral control.

### 3.5. Pure Pursuit Control

**Figure 8:**
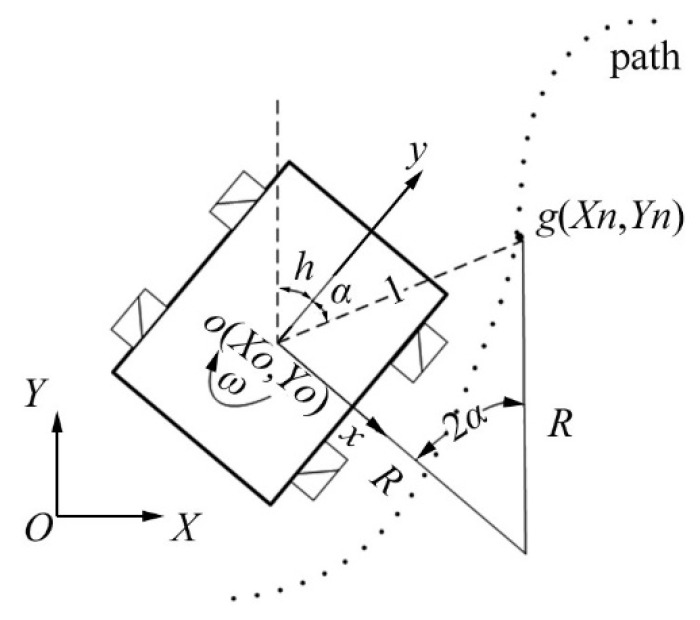
The diagram of pure pursuit control.

The pure pursuit control algorithm is a lateral control method that utilizes the monorail model of a vehicle. The tangent point is the centre of the vehicle’s rear axle, and the tangent line is the longitudinal centre line. By controlling the front wheel turning angle of an automated vehicle, it travels along the arc that passes through the pre-scan point. This method is a geometric tracking-based lateral control method [81,82]. It is robust and still tracks curvature discontinuous paths well, but it is difficult to achieve adaptive pre-sighting distances [83,84], as shown in the diagram of pure pursuit control in Figure 8. Amidi was the first to introduce pure tracking algorithms into path-tracking control for mobile robots. The second DARPA challenge was the first to apply path-tracking control to intelligent vehicles in 2005 [85]. The theoretical approach to the control of autonomous vehicles was further enriched by the optimal pre-targetting control theory proposed by Guo et al. [86].

The pre-scan control’s error feedback is mainly determined by the pre-scan point, which is influenced by the curvature of the road and the speed of the vehicle. In situations where the road curvature is large, the path-following control may be a ‘short-cut’, leading to a significant lateral error. Similarly, when the road curvature changes quickly, the error may fluctuate considerably, causing fluctuations in the control output. To address these issues, multi-point pre-scan control methods have been developed as a means of compensating for the limitations of single-point pre-scans. Symonds [87] proposed a new method of pre-scan, which was to perform a multi-point pre-scan to establish a link between the pre-scan error and the actual front wheel turning angle. Rucco et al. [88] employed a multi-point pre-scan control technique. The method used involved selecting multiple pre-scan points and combining the error feedback quantities from these points to minimize fluctuations in the system output. This is a widely used technique for steering control in autonomous vehicles. While pre-scan control has shown promise in certain scenarios, its overall performance is hindered by model limitations and uncertainties surrounding external disturbances and tire side-bias characteristics.

### 3.6. Linear Quadratic Regulator (LQR) Control

**Figure 9:**
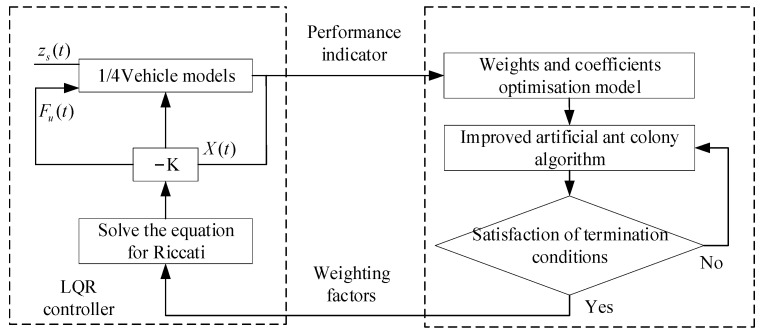
The structure of the controller.

A linear quadratic regulator (LQR) is a type of control system that utilizes state-space equations to create an optimal dynamic controller. The objective function of the controller is a quadratic energy function that takes into account both the system state and the control quantity [89]. The optimal control quantity is determined by minimizing this energy function [90,91]. First, a linear error model is built using the error between the actual vehicle position information and the planned sampling points in each sampling period. Then, by solving the linear quadratic optimization objective function, the optimal solution is obtained to achieve the optimal path-tracking control law acting on the controlled platform of the automated vehicle [92]. Wang et al. [93] introduced an LQR controller that utilizes optimized weighting coefficients. These coefficients were optimized using an improved artificial ant colony algorithm, which results in improved smoothness and operational stability of the vehicle. The whole process of the controller algorithm is shown in Figure 9.

Sun et al. [94] built a control model based on a common bicycle model with the centre of collision as the reference point and proposed a linear quadratic regulator algorithm to design a path-tracking controller to complete tracking of the target path. Wang et al. [95] introduced a combination of a pre-scan strategy and an LQR to regulate the lateral motion parameters of the automated vehicle and explored the effect of the pre-scan distance on the weighting matrix of the controller. Finally, a simulation model was constructed and tested on a real vehicle to verify the controller’s effectiveness. Ni et al. [96] used a four-wheel steering vehicle as the test object to perform lateral motion control. First, the vehicle dynamics equations were constructed, and an error model was established using a pre-sighting strategy. Then, a four-wheel steering vehicle lateral controller implemented in the LQR method was proposed by combining the dynamic model and the error model. Finally, the effectiveness of the algorithm was verified using a simulation model.

According to Ref. [97], the LQR-based lateral controller is prone to overshooting when sudden changes occur in the road curvature. To address this issue, a feedforward control can be incorporated into the road curvature design, resulting in a feed-forward feedback controller that helps to mitigate the overshoot problem and improve the accuracy of lateral control.

### 3.7. Model Predictive Control

Model predictive control (MPC) is a control strategy that utilizes an optimization method to generate a sequence of control inputs by predicting the system dynamics via a model used for control. This method is also referred to as the rolling time-domain optimal control [98,99]. In MPC, only the current time control quantity is utilized as the control input, and the optimal input value is calculated iteratively by continuously updating the optimization interval using a rolling time-domain approach [100,101,102]. Numerical solutions can be obtained for the finite optimization interval, thereby allowing the model prediction to control systems beyond linear systems, in contrast to optimal control. Nonlinear model predictive control has the disadvantage of requiring a significant amount of computation for the numerical solution of nonlinear systems. This results in a large number of operations that must be completed in the control cycle, which can be a drawback. In the context of lateral control in vehicles, when the control cycle is short, it is necessary to use a linear model for model control due to limitations in computing power. Model predictive control algorithms are a more reliable option and maintain high accuracy even in complex environments, as evidenced by previous studies [103,104,105].

**Figure 10:**
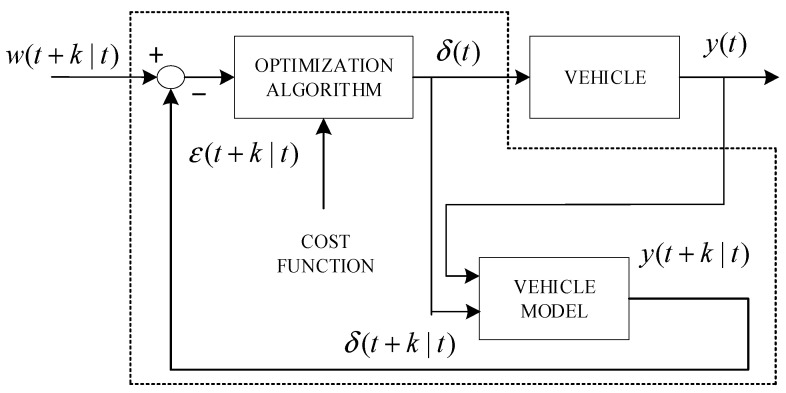
The model predictive control.

Rafaila et al. [106] presented a nonlinear model predictive control (MPC) approach for the autonomous steering of an automated vehicle. The proposed method utilizes a 2-degree-of-freedom bicycle model and a magic formula tire model. At each time interval t, the vehicle model predicts the future lateral position of the vehicle, denoted as y(t + k|t). The corner control signal, δ(t + k|t), is then determined by solving the extreme value of an objective function. This function aims to minimize the difference between the system output and the desired lateral position, w(t + k|t), while also satisfying a predefined constraint. The variables δ(t) = δ(t|t) and y(t) = y(t|t) in Figure 10 represent the front wheel corner input and vehicle lateral position, respectively.

Wang et al. [107] proposed a variable parameter path-tracking control method for a smart connected vehicle, which was based on the MPC principle, and designed a path-tracking controller for a smart connected vehicle. The method used a vehicle model with a 3-degree-of-freedom model; the system was linearized to determine the quadratic objective function of the system, and the matrix form based on the functional form was determined. Subsequently, offline simulations were conducted on both the Carsim and Simulink platforms to determine the appropriate parameters for the path-tracking controller under various typical working conditions. Ultimately, the system was able to select the optimal control parameters based on the actual road shape and vehicle speed information. The information obtained from the vehicle network can help the intelligently networked vehicle to complete automatic steering. Jiang et al. [108] proposed an adaptive path-tracking control approach for intelligent vehicles using the MPC principle. They designed a path-tracking controller and established a path-tracking comprehensive evaluation index based on the total variance method. The path-tracking effect under different road adhesion coefficients was analyzed by simulation, and the relationship curve between the road adhesion coefficient and the optimal vehicle speed was fitted. According to the different vehicle speeds and road curvatures, the optimal time-domain parameters under all typical curve conditions were obtained using genetic algorithm optimization. The longitudinal vehicle speed and road curvature feedback were introduced into the MPC path-tracking controller, which had adaptive time-domain parameters.

The model predictive control (MPC) algorithm is widely recognized for its superior control performance, but it also has some limitations. One of these limitations is that the algorithm requires multiple optimization solutions to be carried out within the predicted time domain. The complexity of the prediction model can limit its solution efficiency and real-time performance, making it challenging to meet real-time performance requirements. Therefore, it is crucial to optimize the prediction model according to specific requirements to achieve optimal performance in different scenarios.

Table 2 presents a comparison of the results of each control algorithm. It is important to note that other control methods were not included in this study. For example, Yoshimoto [109] proposed an automatic steering algorithm that utilizes image processing. The algorithm is designed to eliminate the sum of the lane split line offset and the lateral deviation. The iterative learning control (ILC) [110] has been proposed as a solution to address the problem of optimal control signal design in automated vehicles, which requires tracking a fixed reference trajectory and addressing repetitive disturbances like inclined roads. This approach has been used in automated vehicle tracking control, as demonstrated by Son [111].

The lateral control of intelligent vehicles has demonstrated effective control efforts via the integration of model predictive control algorithms with other control methods. Various control structures, including series and parallel structures, have been implemented. Additionally, independent control of different controllers under varying operating conditions has been proposed. Currently, there are mainly some typical controllers that can enable the system to respond quickly when dealing with large errors, and other controllers can enable the system to track accurately when dealing with small errors. MPCs, for example, are often combined with other control methods to ensure accuracy and stability. Adaptive control thinking and machine learning are combined. The use of Kalman filtering, convex optimization, nonlinear optimization, and other traditional adaptive and machine learning methods optimizes controller parameters, and it can also improve efficiency and ensure control effects for intelligent vehicles. In increasingly complex environments, these controls can still be fast and stable to complete the tracking of the target path. One of the main challenges in tracking control is accurately modelling the dynamics of the entire vehicle, as well as effectively controlling the coupling of the vehicle in both the lateral and longitudinal directions. Additionally, the controller must account for nonlinearity, uncertainty, and external disturbances within the system.

## 4. Summary and Outlook

At present, vehicle motion control methods mainly focus on the design of separate horizontal and vertical controllers by decoupling the horizontal and vertical dynamics. Research on cooperative horizontal and vertical control is limited and at the stage of theoretical analysis. Since a control strategy can be used in practical engineering, as well as the characteristics of horizontal and vertical dynamics correlations, the design methods for the coordinated control strategy need to be further studied in depth.

As the use of autonomous vehicles becomes more widespread, it is becoming clear that a single planning and control algorithm may not be sufficient to meet the demands of every scenario. Currently, multiple control algorithms are utilized to achieve the planning and control of a single scene. In the future, various algorithms will likely be integrated in order to complement the strengths of each other. Additionally, as the number of application scenarios increases, it will be important to improve the generalization ability of the algorithms. This will be a key area of research in the future.

Several countries have already begun implementing intelligent transportation systems to improve their overall transportation infrastructure. This involves utilizing vehicle networking technology to provide real-time and accurate information via vehicle sensor systems and vehicle-to-vehicle and vehicle-to-road communication systems. This increased flow of information will allow for more precise path planning for vehicle lateral control, enabling it to participate in the planning and control of the integrated ‘human-vehicle-road’ transportation system.

The multi-vehicle collaboration of automated vehicles still faces great challenges. The interaction of information leads to an exponential increase in the amount of data, and the requirements for arithmetic power are becoming higher and higher. The undulating terrain causes the attitude of the vehicle to change frequently, which requires a high level of spatial and temporal consistency for the collaborative detection of multiple targets. However, the intelligence of the current intelligently networked vehicles is lower than that of human drivers. Therefore, improving the intelligence of intelligently networked vehicles is an important research direction for the future.

## Figures and Tables

**Table 1:** The planning algorithm comparisons.

Algorithms	Comments
Graph search algorithm	The amount of computation increases dramatically with increasing accuracy and is not suitable for application in complex environments.
Curve interpolation algorithm	Low computational cost and high real-time performance.
Sampling-based approach	The solution speed is fast, but the solution accuracy is not high.
Artificial potential field method	A relatively large advantage is path planning in uncertain dynamic environments, but it is easy to fall into local minima.
Machine learning methods	Fast computational speed and good generalization capability, but a large number of training samples are required.
Numerical optimization algorithms	More efficient solving, but real-time performance needs to be improved.

**Table 2:** Control algorithm comparison.

Algorithms	Comments
PID control	It has the advantages of easy implementation and high stability, but the parameters are difficult to optimize and the control performance is poor.
Robust control	It is highly resistant to interference, but it is difficult to achieve the optimal state.
Sliding mode control	It has a fast response, is insensitive to parameter changes and disturbances, and has simple and reliable control action.
Fuzzy control	It does not require precise system modelling and is fault-tolerant, but relies excessively on rule bases.
Pure pursuit control	It has better robustness but is sensitive to speed changes and prone to overshoot.
Linear quadratic regulator (LQR) control	It utilizes lower cost to obtain better control performance with high practicality, and it has better robustness against the effect of noise.
Model predictive control	It is easy to model, has better system robustness and stability, and can effectively deal with multivariate, constrained problems.

## Data Availability

All data are available within the manuscript.
